# Death Rates

**Published:** 1915-07

**Authors:** 


**A Monthly Review of Medicine and Surgery**

EDITOR AND PUBLISHER, DR. A. L. BENEDICT, 228 Summer St., corner of Elmwood Ave., Buffalo, (Address for all communications. Please make personal and telephone calls before 1 P. M.)

Third, in age, on the western continent. Independent, but supports professional organization. News limited mainly to Western N. Y. and Penn.

Subscription $2.00 a year; $3.00 for two years in advance. Changes and errors in address or failure or duplication of delivery, should be reported immediately.

Yearly Volume 70. JULY, 1915. No. 12

BILLS FOR SUBSCRIPTION FOR THE NEXT VOLUME AND FOR ARREARAGES WILL BE INCLOSED WITH THE AUGUST ISSUE, AS USUAL.

---

### Death Rates.

Some recent, rather acrimonious discussion of death rates, suggests this article. It is obvious that the direct, crude, death rate is obtained by dividing the actual number of deaths in any community by the population. If the numbers are accurately known, the result is accurate. Under existing conditions, the dividend is quite accurately known for any year or month, while the divisor (population) is known only at intervals of 10 or 5 years, if state or police censuses are taken. In a community normally increasing in population, it is obvious that if the known population of the latest census year is used as a divisor for future years, the apparent death rate progressively increases as compared with the real, until the next census figures are obtainable, when it jumps back to its normal.—ignoring the inevitable fluctuations. If, as is now commonly practiced, the rate of increase is estimated, the results may or may not be correct, according as the actual increase does or does not conform to precedent.

As the death rate now has a pretty direct bearing on the commercial and social prosperity of a community, it is obvious that, so far as possible, the crude death rate should be corrected to exclude the influence of deaths of non-residents and, especially deaths due to conditions not existing in the community itself. At present, it is approximately true, at least for New York State, that the large cities, even judged by crude death rates and more significantly when judged by the mortality due to typhoid and other infections, are slightly more healthful than the country, while villages and small cities are, on the whole, apt to show the worst conditions. When we consider that the larger cities are medical and surgical centers, naturally attracting serious cases from the country and that, however skillful the treatment accorded these cases, a disproportionate number of them inevitably die, it is plain that the difference between the true death rates of city and country is greater even than appears from crude death rates. Moreover, any large institution, as for the insane, orphans, the tuberculous, even a prison, has a higher death rate than the general population and, if situated, as many are, in comparatively small communities, tends to increase the crude death rate of that community enormously. In such cases, the question arises how far the prolonged and more or less compulsory residence of the defective population, should be considered a residence in the statistic sense. Again, particularly with regard to typhoid, the question arises whether a given community should be considered responsible for infections or other causes of death contracted while absent from the place of residence but occurring within the boundaries of the community. It is sometimes argued that allowance should not be made for deaths of non-residents, since deaths of residents temporarily absent from home, balance these. In the long run, this is true but it scarcely applies to cities. Excepting tuberculosis—and even then, with due regard for the tendency to return home to die—deaths of absentees are largely due to accident for which the home town is in no way responsible. Moreover, a comparatively small proportion of residents are away from home at any one time and their liability to death is slight, as compared with that of non-residents, seeking the city as a desperate hope of relief from serious medical and surgical conditions.

But, if we once begin to standardize the crude death rate, it is difficult to say when we should stop. Some argue that the average age of the inhabitants of rural districts is higher than that of cities and that, therefore, the death rate is necessarily higher. Conversely, it is a well known paradox of vital statistics that a high birth rate necessitates a high death rate. As is well illustrated by the tables published for the years 1913 and 1914 by the Health News (N. Y. State Dept. of Health) for April 1915, a high birth rate does not necessarily imply a high death rate of the infants of the same year but the first year of life is particularly vulnerable and under the best conditions as yet obtained and maintained beyond exceptionably favorable periods, is about 10%, equalling that of advanced ages and applying to a far greater part of the total population. The very varying rates at which age-mortality decreases and increases, the variations for sex, etc., make it extremely difficult to standardize the death-rate for different populations, even according to well marked differences in the proportions of elderly persons.

In comparing European with American death rates, it is almost universally conceded that allowance should be made for the fact that our population increases very markedly by the increment of adults, more of whom are men than women, and all of whom have been at least nominally inspected as to health. Yet it is questionable and scarcely answerable by statistics, how far this artificial disturbance of growth can be balanced by modifications of the crude death rates. On the one hand, it may be claimed that European countries subject to large emigration, must have relatively high proportions of the infant and senile groups, both of which are highly vulnerable while America has a higher proportion of older children and young and middle-aged adults, with males in slight preponderance, tending toward lower inevitable mortality. On the other hand, there can be no question but that the recent immigrant is subjected to privations, is forced to engage in hazardous occupations, and, if as is often the case, is separated from his family, incurs venereal and other social risks. Within a few years after immigration, this increment to our population reproduces rapidly and under relatively unfavorable conditions, so that a further compensatory increase of death rate occurs. In passing, it may be pointed out that, nevertheless, the United States increases in population very largely by immigration and to a comparatively slight degree by births; also that any estimates of population, death rate, etc., based on average increase in the past, will be very seriously affected for some years to come. The actual fact remains that certain communities of Europe show markedly higher death rates than apparently comparable communities here, while others show rather more favorable results, especially for the later years of life. But, when it comes to balancing the various factors involved and correcting crude death rates to allow for the reciprocal influence of migration, we question whether anyone can mathematically state what the normal rates should be, IF.

It has seldom occurred to statisticians to consider that the same factor of migration applies to contrasts of rural and municipal death rates, in somewhat the same ways and in some very different ways. The tide from the country to city is mainly of young adults and perhaps mainly of males. On the one hand, these intra-national migrants are subjected to no formal inspection, on the other hand, they are subjected to comparatively little of the gross unfavorable conditions of the inter-national migrant. There is a prevalent idea that many of them return to the country—at least to small towns, late in life. They do not tend to reproduce abundantly nor under unfavorable conditions as do the inter-national migrants. Therefore, other things being equal, urban mortality ought to be low as compared with rural. But certain opposed facts must be considered. The gradual increase in "urban" population is by no means due solely to migration of young adults, much less, to any excessive degree of male adults. To a very large degree, it is due to the normal increase in villages which tend to throw them into the arbitrary designation of "urban." It is a great misconception to think that the changes between rural and urban population have been due at any time in our history merely to "flocking to the city." If, even with this allowance, the country were the breeding place and the place of retirement of the aged, it would have a normally higher death rate than the city, on account of the inevitably higher vulnerability at the extremes of life. The actual facts, however, are that, on the one hand, the birth rate of the cities is on the average far higher than that of the country and that, on the other, the city dweller does a good deal of talking about retiring to the country to spend his declining years and very seldom does so while, in many instances, the senile country dweller or the one otherwise very liable to die, goes to some institution or to the home of his children in the city.

All things considered, we are inclined to think that attempts to standardize death rates for purposes of comparison by calculating what the death rate in one community would be, if it had the same kind of population as another, are futile, except that allowances may properly be made for deaths of non-residents due to causes antedating their sojourn and for the inclusion of institutions notably affecting the vulnerability of a relatively large proportion of the population and drawing largely on outside territory.

